# Projected avifaunal responses to climate change across the U.S. National Park System

**DOI:** 10.1371/journal.pone.0190557

**Published:** 2018-03-21

**Authors:** Joanna X. Wu, Chad B. Wilsey, Lotem Taylor, Gregor W. Schuurman

**Affiliations:** 1 Science Division, National Audubon Society, San Francisco, California, United States of America; 2 Natural Resource Stewardship and Science, US National Park Service, Fort Collins, Colorado, United States of America; Universita degli Studi di Napoli Federico II, ITALY

## Abstract

Birds in U.S. national parks find strong protection from many longstanding and pervasive threats, but remain highly exposed to effects of ongoing climate change. To understand how climate change is likely to alter bird communities in parks, we used species distribution models relating North American Breeding Bird Survey (summer) and Audubon Christmas Bird Count (winter) observations to climate data from the early 2000s and projected to 2041–2070 (hereafter, mid-century) under high and low greenhouse gas concentration trajectories, RCP8.5 and RCP2.6. We analyzed climate suitability projections over time for 513 species across 274 national parks, classifying them as improving, worsening, stable, potential colonization, and potential extirpation. U.S. national parks are projected to become increasingly important for birds in the coming decades as potential colonizations exceed extirpations in 62–100% of parks, with an average ratio of potential colonizations to extirpations of 4.1 in winter and 1.4 in summer under RCP8.5. Average species turnover is 23% in both summer and winter under RCP8.5. Species turnover (Bray-Curtis) and potential colonization and extirpation rates are positively correlated with latitude in the contiguous 48 states. Parks in the Midwest and Northeast are expected to see particularly high rates of change. All patterns are more extreme under RCP8.5 than under RCP2.6. Based on the ratio of potential colonization and extirpation, parks were classified into overall trend groups associated with specific climate-informed conservation strategies. Substantial change to bird and ecological communities is anticipated in coming decades, and current thinking suggests managing towards a forward-looking concept of ecological integrity that accepts change and novel ecological conditions, rather than focusing management goals exclusively on maintaining or restoring a static set of historical conditions.

## Introduction

The U.S. National Park Service (NPS), the first of its kind in the world, offers uniquely strong legal and institutional protections that “preserve unimpaired the natural and cultural resources” as mandated by its founding legislation [1]. However, despite protection within the NPS, national park ecosystems face numerous modern threats including invasive species, pollution, regional development and habitat fragmentation, modification of natural processes, and climate change [2]. Among these, climate change warrants particular research attention because its impacts are (1) relatively new and not fully understood, (2) pervasive, and (3) likely to influence impacts of other stressors. Ongoing climate change has brought warmer annual mean temperature, winter lows, and summer maximums [3], such that the vast majority of parks are already at the extreme warm edge of historical conditions [3–4]. Precipitation patterns are also changing [3], and collectively these changes have the potential to modify species assemblages and ecological processes within national parks [5].

Birds are useful indicators of ecological change because they are highly mobile, responsive, and generally conspicuous. They are also popular with visitors to national parks; bird-watching is a $107-billion industry in the U.S. that involves 47 million people annually [6]. Birds are among the most studied taxa, and numerous studies have documented climate change impacts and responses in birds [7], including phenological mismatches in resources and breeding [8], warm-adapted species increasing in abundance relative to cool-adapted species [9–10], and regional endemics declining more sharply with warming [11]. Some populations may already be experiencing direct impacts from changes in the frequency of extreme weather events [12], for example, through elevated mortality during heat waves [13]. A global synthesis of peer-reviewed research suggests that one in five bird species has experienced negative impacts of climate change in some portion of its range [14], including local extirpation [15]. Local extirpation is synonymous with range contraction unless accompanied by expansion (via colonization) elsewhere, in which case the impact would be range shift. Bird range boundaries are already shifting in elevation [16] and latitude [17–18] with rising temperatures and altered precipitation patterns, and over the past 60 years, U.S. bird species’ northern range boundaries have generally expanded while eastern and western boundaries have contracted [19].

Future climate change poses an intensifying threat to many bird species, with global to local implications. A global assessment of climate change vulnerability based on climate change exposure and trait-based measures of sensitivity and adaptive capacity (considering traits such as habitat specialization, lifespan, dispersal capacity, and fecundity) suggests 24–50% of birds are highly vulnerable to climate change [20]. In the U.S. and Canada, a model-based assessment of climate sensitivity suggests 21% of bird species are highly sensitive to climate change, such that they may lose half of their climatically suitable ranges by mid-century [21]. In the Great Lakes region of North America, 26% of 46 migratory species were classified as vulnerable to climate change when their full annual cycle was considered [22]. Locally, 36% of 358 birds in California were classified as vulnerable to climate change with 72% of the 29 state or federally listed species among them [23]. A changing climate will impact a park’s suitability for species, with corresponding effects on community composition and relative abundances. Climate suitability for a species is characterized using ecological niche models, which correlate species occurrence data with environmental covariates including climate [24]. Model predictions can be transferred into geographic space to map species distributions under projected future climate [25]. Estimates of climate suitability may correlate with relative abundance [26–27], and changes in climate suitability track population trends over time [28]. Thus, projections of changing climate suitability can provide insight into how populations and assemblages may respond to future climate change.

The threat of climate change to birds is not only a novel and pervasive challenge to a charismatic taxon, but also to traditional conservation goals and the expectation that protected areas can retain historical ecological components and processes. Understanding the future of birds in parks can inform long-term park management and public engagement. Here, we characterized projected changes in climate suitability for 513 species of birds across 274 national parks in the United States, grouping species within each park based on their projected trends in climate suitability. We quantified potential species turnover across parks and examined patterns at regional and continental scales. Finally, we classified parks into trend groups based on projected change in community composition.

## Methods

### Study sites

**Fig 1:**
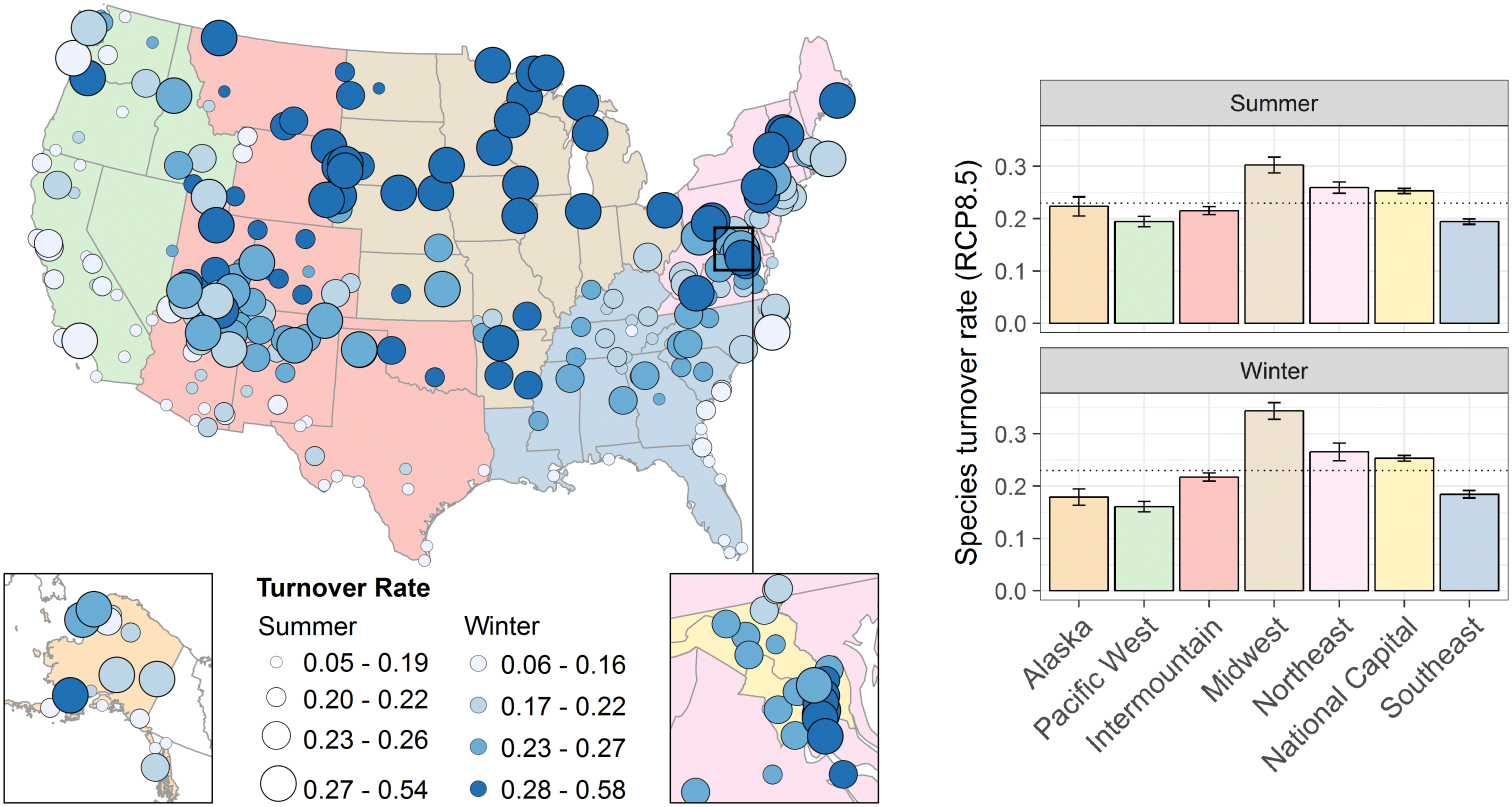
Projected species turnover from the early 2000s to mid-century across seven NPS geographic regions and 274 U.S. national parks. Bray-Curtis turnover rates under RCP8.5 are calculated under the assumption that all potential extirpations and colonizations are realized, with 0 being no change and 1 being complete turnover. Circle sizes represent rates in summer, and colors represent rates in winter. Breaks in classes are based on quartiles. Alaska is shown in the inset on the left and the National Capital region is shown in the inset on the right. The chart on the right shows the mean and standard error of the mean turnover index by NPS geographic region, and the dotted lines show the mean turnover index across regions in both summer (0.23 ± SE 0.004) and winter (0.23 ± 0.006). Analysis of variance indicated significant difference among regions in summer (F_*(6*, *267)*_ = 13.96, *p* < 0.0001) and winter (F_*(6*, *267)*_ = 26.25, *p* < 0.001).

Our analysis encompassed 277 national parks in the continental U.S. that fall under the NPS Inventory and Monitoring Program’s natural resource park designation and represent 49 of 50 U.S. states, with the exception being Hawai‘i. A total of 274 national park management units (hereafter referred to as “national parks” or “parks”) remained after grouping together those parks that are typically managed as a single unit (i.e., Sequoia and Kings Canyon National Parks, and three park units that collectively constitute the Roosevelt-Vanderbilt National Historic Site). The studied parks represented seven NPS geographic regions (Fig 1), with the number of parks in each region reported in parentheses, Alaska (16), Pacific West (39), Intermountain (82), Midwest (36), Southeast (44), National Capital (18), and Northeast (39), and spanned 25° to 68°N latitude and 68° to 164°W longitude.

### Climate suitability projections

Projections of future climate suitability for each species were based on a suite of previously published species distribution models for North American birds [21,29]. Models of summer and winter distributions were trained on observations from the North American Breeding Bird Survey [30] and Audubon Christmas Bird Count [31] using 17 bioclimatic variables as predictors (S1 Table). We took these seasonal distribution models [29] and updated their projections for two future greenhouse gas (GHG) concentration trajectories—RCP2.6 (Representative Concentration Pathway) and RCP8.5—representing the low and high extremes of potential future greenhouse gas trajectories. RCP2.6 is the most stringent mitigation scenario, whereas RCP8.5 most closely approximates a continuation of the current pathway of rising emissions [32]. Four CMIP5 (Coupled Model Intercomparison Project) general circulation models (GCMs; CanESM2, CESM1/CAM5, HadGEM2-ES, MIROC-ESM) were used to capture the extremes and average climate warming possibilities across North America [33]. A 10-km buffer was applied to each park to match the spatial resolution of the species distribution models (10 x 10 km) [29], and climate suitability for each park was taken as the average of all cells encompassed by the park and buffer. Climate suitability was estimated for the present (2000–2010), and projected to 2041–2070 (hereafter, mid-century) across the continental U.S. and Canada. We present results averaging climate suitability values across GCMs between the present and mid-century, considering each emissions trajectory separately. We present results primarily from RCP8.5 (hereafter, the high-emissions pathway) as the scenario most consistent with current GHG emissions rates, and make comparisons to RCP2.6 (hereafter, the low-emissions pathway) as a contrasting best-case scenario for GHG emissions reductions [33].

### Park analyses

We treated parks as the unit of analysis to assess projected changes in climate suitability. For a species to be considered for analysis in a particular park, climate in the park had to be suitable for the species either in the present or by mid-century. The true skill statistic (TSS), which maximizes true presences and true absences in species distribution models and is unaffected by prevalence [34–35], was used to convert climate suitability values of each species into suitable/unsuitable.

**Fig 2:**
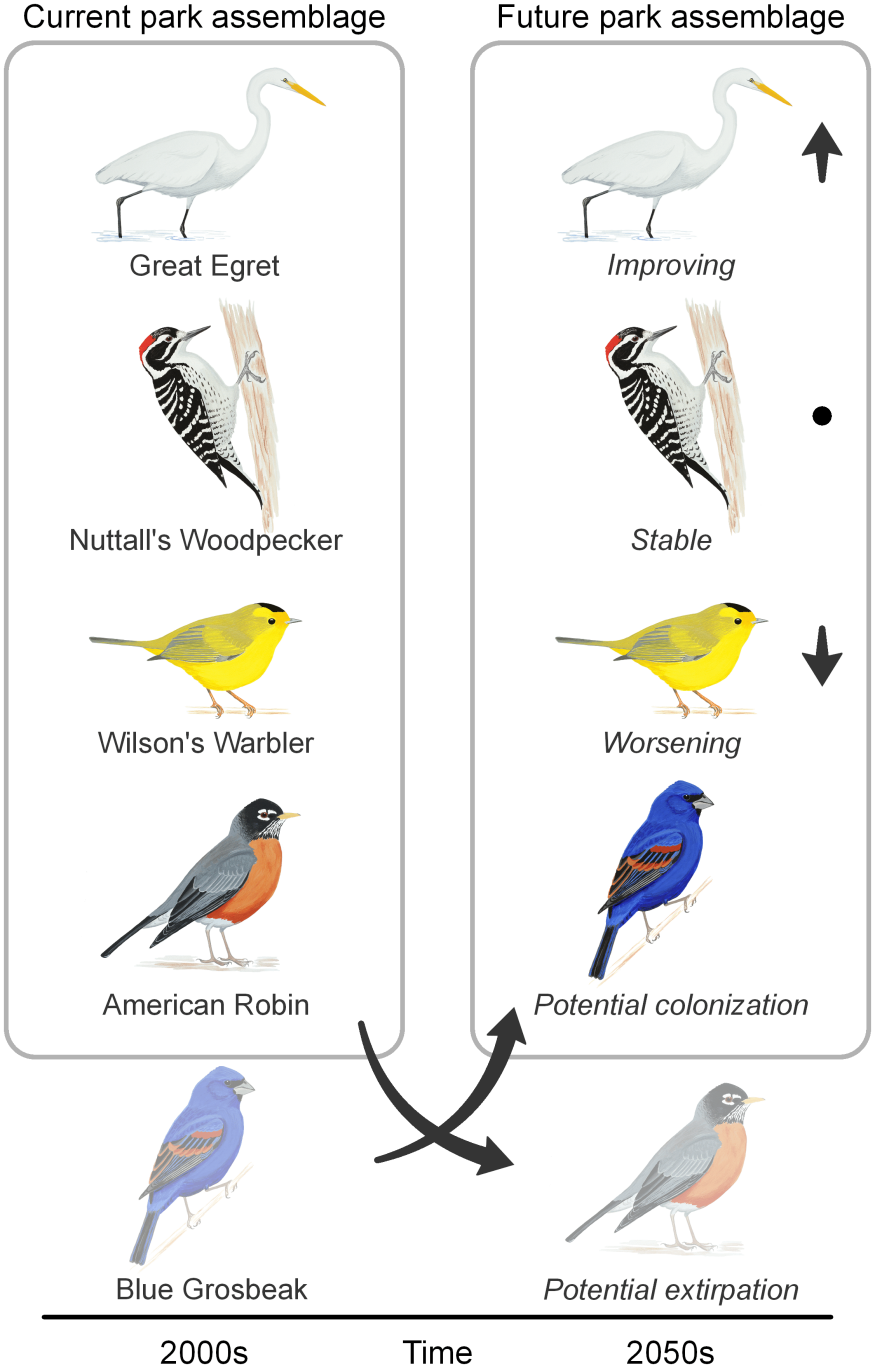
Example of projected bird assemblage changes by mid-century at Golden Gate National Recreation Area. Under RCP8.5 in summer, climate suitability is projected to improve for the Great Egret (*Ardea alba*), remain stable for the Nuttall’s Woodpecker (*Picoides nuttallii*), and worsen for the Wilson’s Warbler (*Cardellina pusilla*). Climate suitability is at risk of disappearing for the American Robin (*Turdus migratorius*), potentially resulting in extirpation from the park. Although the Blue Grosbeak (*Passerina caerulea*) is not currently found in the park, climate is projected to become suitable for this species, potentially resulting in local colonization. Bird illustrations by Kenn Kaufman.

We characterized the implications of future climate change for species within each park based on two criteria: (1) an estimated trend in climatic suitability and (2) whether climate suitability crosses a threshold, suggesting a higher potential for colonization or local extirpation (Table 1). For the first criterion, we fit a linear regression (*y* = *β*_*0*_ + *β*_1_*year) with suitability values (dependent variable) as a function of time. Time periods (independent variable) were the present, 2011–2040, and 2041–2070. Predictions to the intermediate time period were included to improve estimates of trend but are not presented as results. The four suitability values (one for each GCM) capture the variation in projected climate suitability at each time period, and regression finds the average trend across GCMs and over time. To determine the direction and significance of the change in climate suitability over time, we used the coefficient (*β*_*1*_) of the year term to assess whether the slope of each linear model was significantly different from zero. Climate suitability for each species within each park was characterized as improving, stable, or worsening (Table 1; Fig 2). For the second criterion, we assessed whether climate suitability values cross the TSS-derived suitability threshold in either direction: climate that transitions from unsuitable to suitable equates to potential colonization, and climate that transitions from suitable to unsuitable equates to potential extirpation (Table 1).

**Table 1:** Change in climate suitability between the present and mid-century. Each species within each park was classified into one of the following trends.

Classification	Description
Improving	Climate suitability shows a significant positive trend over time; climate conditions are projected to improve
Stable	Slope of change in suitability is not significantly different from zero; no change in climate suitability
Worsening	Climate suitability shows a significant negative trend over time; climate conditions are projected to worsen
Potential extirpation	A subset of the worsening trend where climate suitability is at risk of disappearing (i.e., future modeled suitability falls below the species-specific suitability threshold at present), potentially resulting in extirpation from the park
Potential colonization	Climate conditions are unsuitable at present but are projected to improve sufficiently to become suitable (i.e., future modeled suitability exceeds the species-specific suitability threshold at present), potentially resulting in local colonization

We explored how species assemblages may change in each park over time (i.e., species turnover) by generating a species list for each park for the present and future time periods. We assumed that climate conditions becoming suitable or unsuitable for a species—i.e., potential colonization and extirpation—translate to realized colonization or extirpation. To quantify species turnover, we calculated the Bray-Curtis dissimilarity index within each park, season, and emissions pathway using vegdist from the R package vegan [36]. Differences in climate suitability, potential colonization and extirpation, and species turnover among seasons were compared with a *t*-test where data met normality assumptions, and a Wilcoxon test where they did not.

We also identified species in each park for which conditions are currently only suitable in summer and are projected to become suitable in winter. These species may increasingly find conditions suitable to remain in those parks year-round.

We explored latitudinal trends in climate suitability, using park as the sampling unit. Simple linear regressions were used to analyze potential colonization and extirpation, and species turnover rates (dependent variables) as a function of latitude (independent variable). Parks in Alaska were removed from this analysis due to the data gap in the higher latitudes falling within Canada.

**Fig 3:**
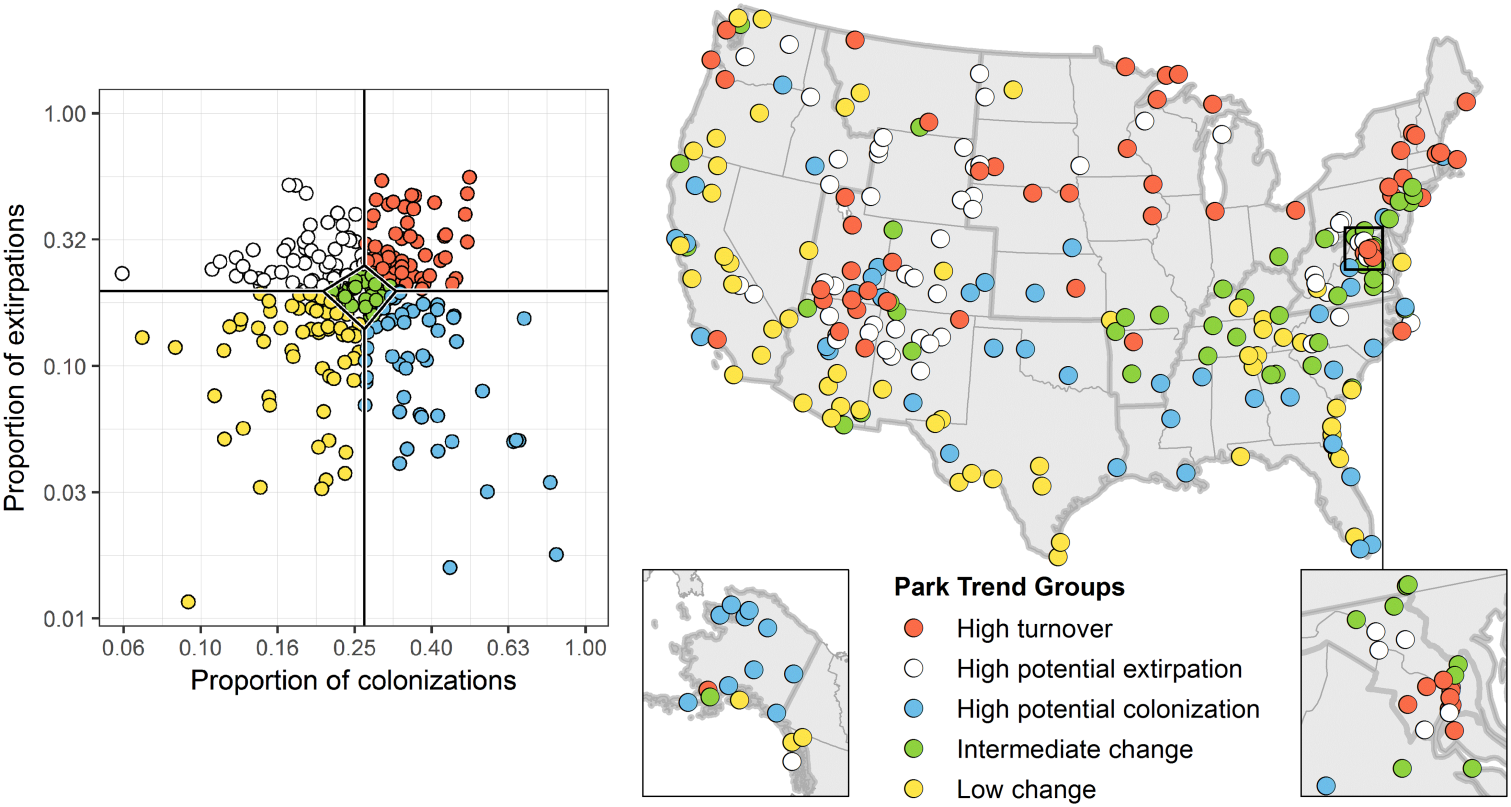
Classification of 274 U.S. national parks into trend groups based on the proportion of potential colonizations and extirpations. Each circle represents a park. The median proportion of colonizations and extirpations across parks under RCP8.5 in summer (represented by solid vertical and horizontal lines in the plot) were used to classify parks into all trend groups except intermediate change. The upper and lower quartiles of each axis (represented by the diamond in the center of the plot) mark the boundaries of the intermediate change group. Alaska is shown in the inset on the left and the National Capital region is shown in the inset on the right.

To understand how projected changes (i.e., colonization and extirpation) in individual parks compare with other parks in the system, we classified parks into relative park trend groups based on the ratio of projected colonizations to extirpations in summer under RCP8.5 following Hole et al. [37]. Plotting projected colonizations and extirpations against each other for all parks, we divided the area of the graph into five sectors based on quartiles (Fig 3). Each park was then classified into one of five categories according to the sector on the graph into which it falls: high turnover, high potential colonization, high potential extirpation, intermediate change (for parks within a quartile of the median along the two axes), and low change (Fig 3).

### Regional analyses

Differences in climate suitability, potential colonization and extirpation, and species turnover among NPS regions were explored with analysis of variance (ANOVA). We checked for heteroscedasticity, given the unequal numbers of parks in each region, and proceeded with ANOVAs since we did not find it to be an issue. All analyses were performed in R version 3.3.2 [38].

## Results

Across the 274 national parks, 513 species (360 species in summer and 396 in winter) were included in this analysis (see S2 Table for projected trends of all species in all parks). The mean number of species included per park and emissions pathway in summer was 102.9 (range 66–153) and in winter was 142.9 (range 29–268). Results for potential colonizations and extirpations are presented in the main text, whereas improving, stable, and worsening trends are summarized in S1 Appendix.

### Potential colonizations more common than potential extirpations

**Fig 4:**
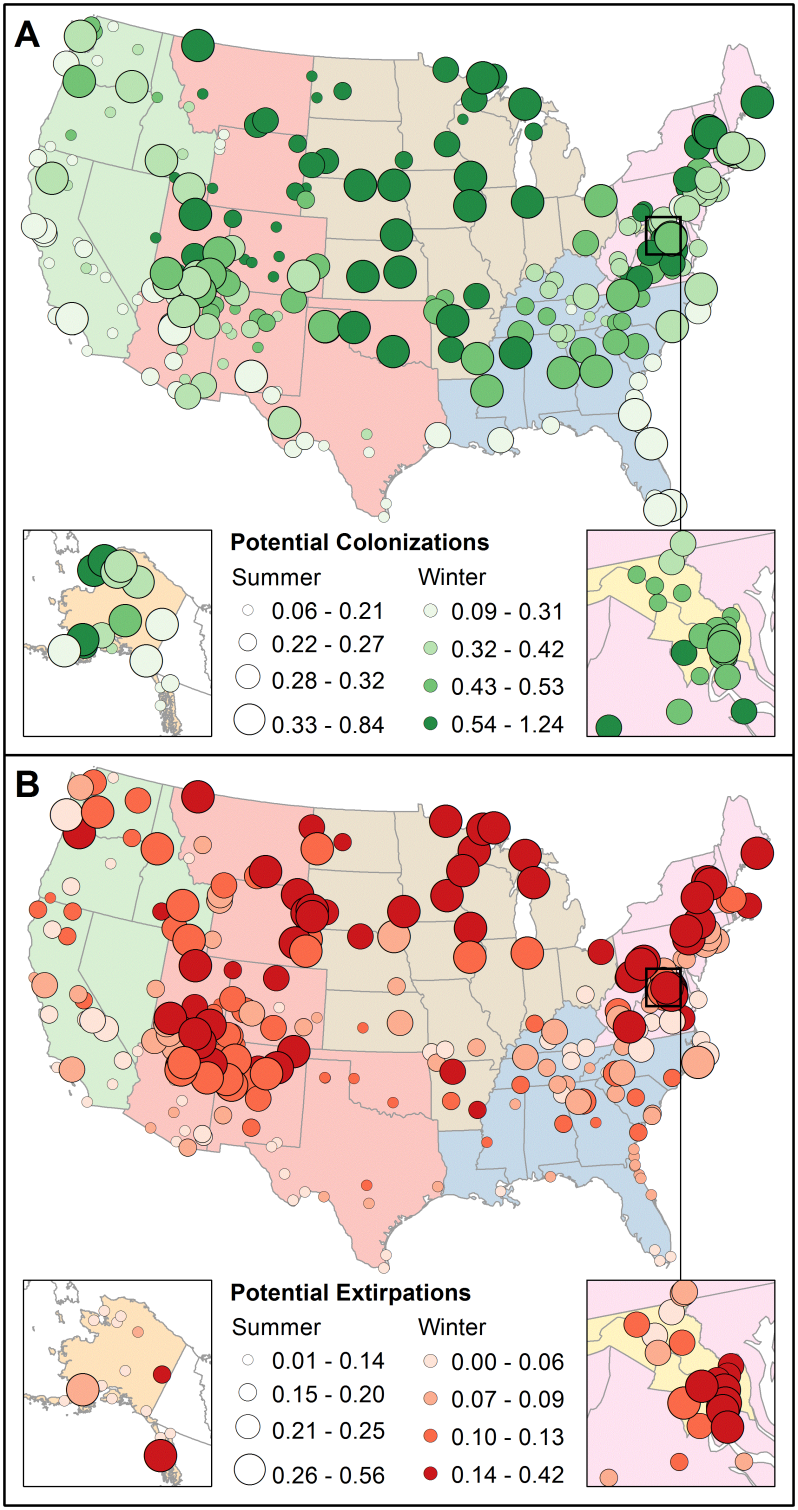
Projected species composition changes from the early 2000s to mid-century across 274 U.S. national parks. Potential (A) colonizations and (B) extirpations in summer and winter under RCP8.5 are shown as a proportion of the current total number of species. Circle sizes represent proportions in summer, and colors represent proportions in winter. Breaks in classes are based on quartiles. Alaska is shown in the inset on the left and the National Capital region is shown in the inset on the right.

Trends in projected future climate suitability suggest that parks could support more bird species by mid-century than they do today in both summer and winter (Table 2; Fig 4). Under RCP8.5, parks are projected to see an average of 22.5 ± 0.4 potential species colonizations and 17.4 ± 0.6 potential species extirpations per park in summer; and 42.1 ± 0.7 potential species colonizations and 10.3 ± 0.3 potential species extirpations per park in winter. Species richness within parks would increase in the future if all projected climate gains in summer (mean ratio of potential future to current species richness under RCP8.5 = 1.07:1) and winter (mean ratio = 1.34:1) were realized. Under RCP8.5, the number of parks where potential colonization represents >25% of their current species count outweighs the number of parks where potential extirpation represents >25% in both seasons (Table 2). The degree of projected change was less drastic under RCP2.6, but nevertheless potential colonizations exceeded potential extirpations in more than 60% of parks, regardless of season or emissions pathway (Table 2).

**Table 2:** Potential changes in bird assemblages by mid-century, by emissions pathway and season, as measured by (1) average ± SE of Bray-Curtis dissimilarity index across parks, average ± SE of (2) the proportion of potential extirpations and (3) potential colonizations across parks, (4) count and percent of parks with more than 25% extirpations, (5) count and percent of parks with more than 25% colonizations, and (6) count and percent of parks where the number of potential colonizations exceeds potential extirpations.

RCP	Season	Bray-Curtis index	Potential extirpation	Potential colonization	Parks with >25% extirpation	Parks with >25% colonization	Parks where colonizations > extirpations
8.5	Summer	0.23 ± 0.004	0.20 ± 0.006	0.28 ± 0.007	68 (24.8%)	151 (55.1%)	195 (71.2%)
8.5	Winter	0.23 ± 0.006	0.11 ± 0.004	0.45 ± 0.013	12 (4.4%)	226 (82.5%)	274 (100%)
2.6	Summer	0.15 ± 0.003	0.14 ± 0.004	0.17 ± 0.005	23 (8.4%)	33 (12.0%)	171 (62.4%)
2.6	Winter	0.15 ± 0.004	0.07 ± 0.003	0.26 ± 0.008	5 (1.8%)	118 (43.1%)	264 (96.4%)

If model predictions of colonization and extirpation were realized, a national park could expect a 23% change (as measured by the Bray-Curtis dissimilarity index), on average, in its bird species assemblage between today and mid-century under RCP8.5 (Table 2; Fig 1). Under RCP2.6, projected changes in potential colonization and extirpation and species turnover are less drastic but the patterns are the same (Table 2).

With a warming climate, bird species may increasingly find suitable conditions year-round in parks where those conditions currently occur only in summer. Under the high-emissions pathway, an average of 7.1 ± 0.2 species per park (range 0–21) might find suitable conditions year-round. This number drops to 4.8 ± 0.2 species (range 0–18) under the low-emissions pathway, which is significantly lower than the high-emissions pathway (*W* = 50660, *p* < 0.0001).

### Seasonal differences

Comparing the two seasons, the proportion of potential colonizations in winter, on average, exceed those in summer (Table 2; *W* = 16932, *p* < 0.0001), and proportion of potential extirpations in summer exceed those in winter (Table 2; *W* = 60632, *p* < 0.0001). The ratio of the average proportion of potential colonizations to extirpations is 4.1 in winter and 1.4 in summer (Table 2). On average, 84.3% of potential colonizations would have to be realized to exceed potential extirpations in summer, whereas only 25.4% of potential colonizations would have to be realized to exceed potential extirpations in winter (mean ratio across parks of number of potential extirpations to colonizations; S4 Table).

### Latitudinal trends

**Fig 5:**
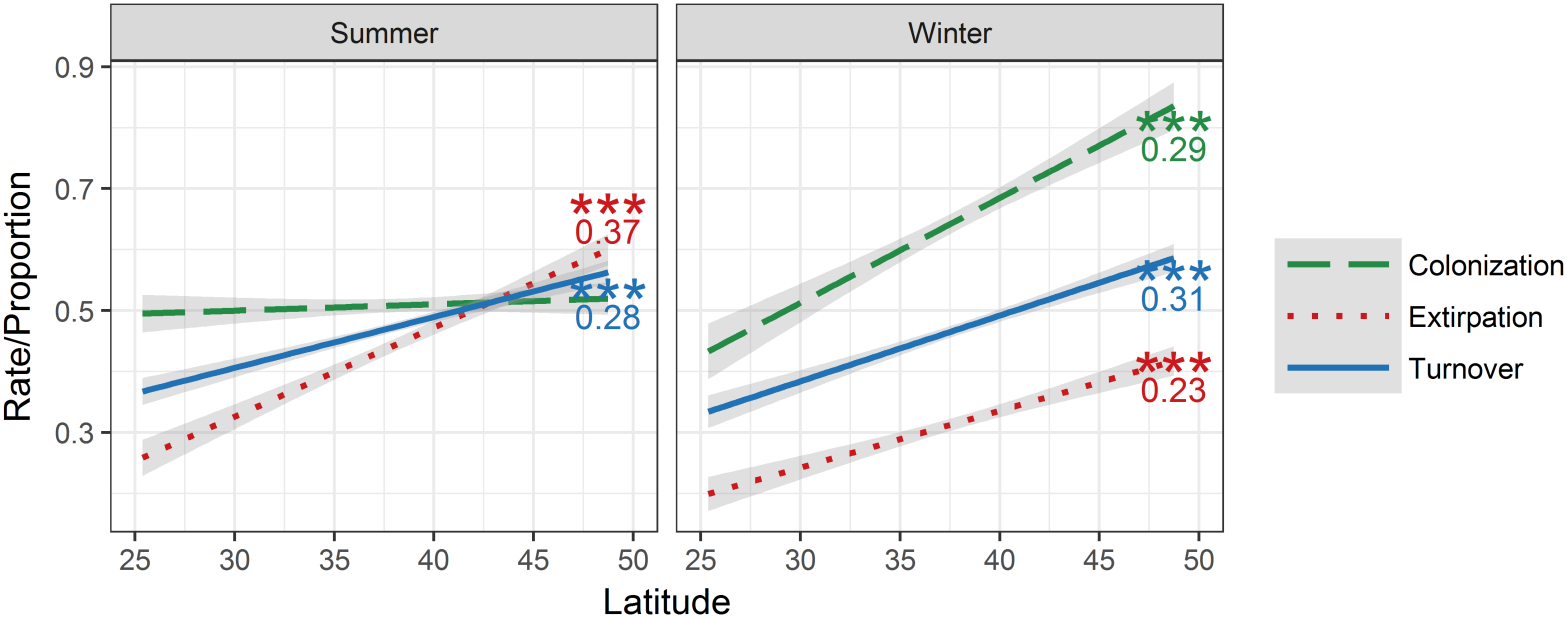
Relationships of the proportion of potential colonizations, extirpations, and turnover rate to latitude. Rates/proportions between the present and mid-century under RCP8.5 in summer and winter. Significance of the regression fit is denoted by “***” where *p* < 0.001, and *r*^*2*^ values are shown next to each curve where significant.

Potential colonizations and extirpations vary with latitude (Fig 5). Under RCP8.5 the proportion of potential extirpations is positively correlated with latitude in summer (*r*^*2*^ = 0.37, *p* < 0.0001) and winter (*r*^*2*^ = 0.23, *p* < 0.0001) in the contiguous U.S. The proportion of potential colonizations is positively correlated with latitude in winter only (*r*^*2*^ = 0.29, *p* < 0.0001). Species turnover is also significantly positively correlated with latitude in summer (*r*^*2*^ = 0.28, *p* < 0.0001) and winter (*r*^*2*^ = 0.31, *p* < 0.0001; Fig 5) in the contiguous U.S.

### Park trend groups

There are 58 parks in the high turnover trend group, 52 parks in the high potential colonization group, 56 parks in the high potential extirpation group, 46 parks in the intermediate change group, and 62 parks in the low change group (Fig 3). The distribution of park trends varies by geographic region (S1 Fig). In summer, the most persistent regions—i.e., the ones with the highest proportion of parks in the low change category—are the Southeast and Pacific West, whereas the Midwest, Northeast, and National Capital regions have more parks with high turnover.

### Regional analyses

The proportions of potential colonizations and extirpations vary by NPS region in both summer and winter (all ANOVAs *F*_*(6*, *267)*_ > 5, *p* < 0.0001; Fig 4). Across all regions under RCP8.5, the number of potential colonizations was equal to or greater than potential extirpations (S3 Table). Species turnover differs significantly by NPS region for both summer (*F*_*(6*, *267)*_ = 13.96, *p* < 0.0001) and winter (*F*_*(6*, *267)*_ = 26.25, *p* < 0.0001; Fig 1). In summer, we found that parks in the Midwest (*W* = 7240, *p* < 0.0001), Northeast (*W* = 6802, *p* = 0.006), and National Capital (*W* = 3368, *p* = 0.009) had significantly higher turnover rates, and parks in the Pacific West (*W* = 3369, *p* = 0.002) and Southeast (*W* = 4037, *p* < 0.001) had significantly lower turnover rates than the mean of parks across the U.S. In winter, parks in the Midwest (*W* = 8169, *p* < 0.0001) and National Capital (*W* = 3379, *p* < 0.01) had had significantly higher turnover rates, and parks in the Pacific West (*W* = 2915, *p* < 0.001), Alaska (*W* = 1500, *p* = 0.03), and Southeast (*W* = 4277, *p* < 0.01) had significantly lower turnover rates than the mean across the U.S.

## Discussion

### Increasing importance of national parks under climate change

The U.S. National Park System, one of the world’s premier protected area systems, will likely be increasingly important to the conservation of birds in the face of climate change. Potential colonization exceeds potential extirpation in more than 60% of parks under both emissions pathways, and if projected extirpations and colonizations were realized, the average park would have 29% more species in winter and 6% more species in summer. While not assessed directly here, the likelihood of these projections occurring may be higher for birds than for other taxa. Birds are highly mobile, making the prospect of range expansion more realistic [39]. Many species are also strongly responsive to climatic factors, with distributions that shift from year to year in response to interannual variation in weather [12,40]. Of course, colonization requires not just suitable climate, but also suitable habitat. Vegetation, in particular, may lag behind, or fail to follow, climate, such that species dependent on mature trees may not find suitable habitat until and unless that vegetation can establish itself [41]. Alternatively, some birds may colonize new regions by showing behavioral plasticity, a form of phenotypic plasticity in which a species demonstrates the ability to change behavior in response to climatic or other drivers of environmental change [42–43]. Phenotypic plasticity may allow a species, for example, to switch to newly encountered species and vegetation types for foraging and nesting, though with potential biotic costs (e.g., reduced nest success [44]). Regardless of the mechanism, colonization in nearly all compass directions along range boundaries has already been observed across bird species [45].

Therefore, many of the potential colonizations of national parks projected above are likely to occur, or are already occurring. The Blue-gray Gnatcatcher (*Polioptila caerulea*), which has expanded north significantly in recent decades [46], is an example. It did not breed in South Dakota in 2001, but was detected breeding for the first time in southwestern South Dakota (Badlands and Wind Cave National Parks) in 2014 and 150 km northwest in northeastern Wyoming (Devils Tower National Monument) in 2015 (pers. comm. K. Gallo). Also of note here is the relatively small proportion (<3%) of introduced species contributing to potential colonization projections (S4 Table), perhaps because most introduced birds, being highly mobile, have realized their niche and/or are naturalized and are no longer expanding their range. Of the nine introduced species in the analysis, only one, the Eurasian Collared Dove (*Streptopelia decaocto*), is known to be expanding its range [47].

### More potential colonization in winter

In winter, approximately one in four potential colonizations would need to occur, on average, to exceed potential extirpations, making national parks more likely to see an increase in species richness in a changing climate in winter than in summer (when seven of ten potential colonizations would need to occur). This may be because winter cold is more of a limiting physiological factor on species distributions [18] than summer heat [48], allowing more colonizations in winter as temperatures increase. Winter months have experienced greater changes in climate (primarily warming temperatures) than summer months [49] and birds have responded by shifting their wintering distributions north [50]. Higher proportions of species showed northward shifts in winter (58%, [50]) than summer (23%, [19]), with warm-adapted species increasingly dominating wintering landbird communities [18]. Migratory species that are already present in summer are perhaps the most likely candidates for winter colonization. Climate change has also already resulted in shorter migration distances for some species [51]. The presence of a species throughout the winter months may result from partial migration strategies, in which individuals cease to migrate, or from chain migration patterns, where individuals from elsewhere in the distribution replace breeding individuals during winter [52]. Or, complete residency could result if selective pressures for shortened migration persist as expected under climate change [53].

### Regional and latitudinal patterns

Projected future changes in community composition varied by region and latitude. In particular, the Southeast and Pacific West had low rates of projected turnover, whereas the Midwest, National Capital, and Northeast had high rates of projected turnover, in both summer and winter. These patterns are consistent with projections of future temperature and precipitation extremes across the U.S. under climate change, which identify more change at higher latitudes in the contiguous U.S., particularly around the Great Lakes, and less change along the Southeast, Gulf, and Pacific Coasts [54]. Low projected turnover in the southeastern U.S. may reflect the impacts of a documented “warming hole” in that region in which both anthropogenic and natural forces combine to reduce warming relative to other regions [55], while high rates of change in the Midwest reflect high projected climate velocities in the mid-continent [56]. Climate velocity, the speed needed to track constant climate conditions, is typically highest in regions with low topographic relief. Similarly, projected biotic velocity, a species-specific measure that reflects the speed required to track constant climate suitability, is high on average for birds in the Midwestern U.S. [57]. The Midwest region is also under pressure from land-use change [58], increasing the value of national parks, national wildlife refuges, and other protected areas for the natural habitats they provide. Climatic connectivity, the ability of species to move through the landscape in order to track constant climate, is lowest in the Midwestern U.S. due to the overall degree of land-cover modification and paucity of natural habitats [59].

Higher latitudes in the contiguous U.S. (~40–49°N) had significantly higher rates of potential extirpation and projected turnover in summer, and higher rates of potential colonization, potential extirpation, and projected turnover in winter. Consistent with findings at the continental scale [21], projected changes in community composition are highest at higher latitudes, and are driven by northward shifts in climate suitability and differences among regions, specifically relatively high projected change in the Midwest and Northeast regions and low change in the Southeast.

An underrepresentation of Mexican bird species in this study may contribute to low projected change at southern latitudes (results not shown). However, significant latitudinal trends in rates of potential colonization and turnover remained after removing from the analysis southern parks likely to experience colonizations from Mexico and Central America. This finding suggests the latitudinal trend is robust to data limitations in the southern U.S.

### Management implications

Protected area networks such as the U.S. National Park System are the focus of landscape-scale conservation [60]. Our analysis reinforces that parks are critical for bird conservation and likely to increase in value with climate change. Furthermore, park trend groups can associate patterns in potential colonization and extirpation across the National Park System with broad suggested climate change adaptation strategies [37] to increase the future effectiveness of the system as a conservation network for birds as well as plants and other wildlife. The assignments of parks to trend groups are relative, such that suggestions for one site consider the degree of projected change elsewhere, and thus can help each park contribute coherently to the goals of the entire park system (S5 Table). Climate-informed conservation strategies focus on increasing habitat connectivity to facilitate movement [59] as well as habitat restoration, protected area expansion, and disturbance-regime management [37,61–62]. These actions might promote resistance (i.e., holding back climate-induced changes to protect highly valued resources), resilience (i.e., improving the capacity to recover after disturbances related to climate change), or facilitation (i.e., facilitating the transition to new ecological conditions) [63]. Management and conservation actions within parks that fall in the low and intermediate change groups can best support landscape-scale bird conservation by emphasizing habitat restoration, maintaining natural disturbance regimes, and reducing other stressors. Parks within one of the three high change groups (high turnover, colonization, or extirpation) can do so by focusing on actions that increase species’ ability to respond to environmental change, such as increasing the amount of potential habitat, managing the matrix surrounding the park to improve connectivity, managing the disturbance regime, and possibly more intensive management actions, such as captive breeding, reintroduction, and translocation (S5 Table). Monitoring to identify changes in bird communities will facilitate the implementation of appropriate management responses. The NPS Inventory and Monitoring Program [64] provides a monitoring framework for validating climate change projections and informing adaptive management in response to climate change. To maximize success, these conservation actions should be implemented within the context of landscape design, a partnership-driven process that aligns conservation goals with stakeholder values and participation [65].

### Caveats

Ecological niche model projections include many inherent uncertainties [24] relevant to this study. Significant changes in climate suitability, as measured here, will not always result in a species response for multiple reasons, including biotic interactions known to both inhibit and facilitate species’ colonization and extirpation [66]; species’ evolutionary adaptive capacity (i.e., the ability to evolve), life history traits [67] and phenotypic plasticity (e.g., behavioral adjustments [68]) mediating responses to climate change; and absence of ecological processes (e.g., that create/maintain habitats or impact demography) in the projections [41,69]. Finally, the TSS-based threshold used to distinguish between improving/worsening conditions and potential colonization/extirpation was selected to balance errors of omission and commission in the models themselves, but results in some imperfections [70–71] and is not the only thresholding method. Ultimately, monitoring is the only way to validate these projections and should inform any on-the-ground conservation action.

### Conclusion

Birds are early responders to climate change and are easily observed and recorded; their responses, including range shifts and behavioral plasticity, are already widely documented [7,72]. National parks, which have an average projected turnover of ~20%, will likely experience considerable change in bird communities. Proactive action is essential to safeguard the existing investment in conservation represented by the U.S. National Park System, as well as to preserve the full complement of management options for the future [62]. A recent re-examination of the Leopold Report, a seminal guide to resource management in national parks, recognizes the importance of “stewardship for continuous change” in preserving natural resources for future generations, and stresses preserving ecological integrity (i.e., “the quality of ecosystems that are largely self-sustaining and self-regulating”) rather than historical conditions [73]. Managers thus face three choices in response to ongoing climate change and associated ecological responses—resist, accommodate, or actively direct ecological change toward specific new desired conditions [4]. Effective conservation in the face of climate change will require landscape-level thinking (including consultation with regulatory bodies such as the U.S. Fish and Wildlife Service in cases of federally protected species) that applies all of these approaches and allows species to persist or track climate. Projections of potential avian community change along with the management recommendations presented here provide a roadmap for informing adaptive management across the network of national parks in the United States.

## Supporting information

S1 Appendix Trends of improving, stable, and worsening species across 274 U.S. national parks. Results for seasonal differences, latitudinal trends, and regional differences are presented for improving, stable, and worsening species.
(DOCX)

S1 Fig Breakdown of park trends by NPS geographic region in summer under RCP8.5. The total number of parks in each region is show above each bar. The Midwest, National Capital, and Northeast regions have the highest proportion of high-turnover parks, whereas the Pacific West and Southeast have the highest proportion of parks with low change. The Intermountain region has a more even mix of park trends. Alaska parks are primarily in the high potential colonization class.
(TIF)

S1 Table Bioclimatic variables used as predictors for species distribution models, and their contributions (averaged across all species) to model fits in summer and winter. Adapted from Table 2 in Distler et al. [29]. A full table of variable contributions to each species’ distribution model can be found in S8 Table in Langham et al. [21].
(XLSX)

S2 Table Climate suitability projections for all species and park combinations by season and emissions pathway (RCP). Climate suitability is a mean across the entire national park’s jurisdiction, except Channel Islands National Park, which excludes a small area of park property on the mainland.
(XLSX)

S3 Table Potential colonizations and extirpations by NPS region, emissions pathway, and season. Means ± 1 SE of the number of species potentially colonizing (No. Col.) and potentially being extirpated (No. Ext.) are shown. Asterisks and signs indicate regions where the mean number of potential colonizations or extirpations in a park is significantly higher (*+) or lower (*-) than the mean across all parks. Colonizations exceeded or were not statistically different from extirpations across regions and emissions pathways. The mean proportions of colonizations and extirpations are shown in Table 2.
(XLSX)

S4 Table Park-specific turnover, colonizations, and extirpations projections. Results for all 274 parks by season and emissions pathway for park trend group (only calculated for RCP8.5, summer), Bray Curtis Turnover Index, current and future species richness (including introduced species), number of potential colonizations (No. Col.) and extirpations (No. Ext.), and number of potential colonizations (Introduced Col.) and extirpations (Introduced Ext.) accounted for by introduced species.
(XLSX)

S5 Table Potential management goals and activities for parks, organized by trend group. Management activities are aimed to optimize the future effectiveness of the U.S. National Park System as a conservation network that fosters bird species persistence in the context of climate change-driven range shift and avian community change. Adapted from Table 1 in Hole et al. [37].
(XLSX)

S6 Table All input data (climate suitability values) across species and parks in summer. (7Z)

S7 Table All input data (climate suitability values) across species and parks in winter. (7Z)

## References

[1] U.S. Congress. The National Park Service Organic Act. National Park Service, 39 Stat. 535, 16 U.S.C. Sect. 1–4 1916.

[2] Wade AA, Hand BK, Kovach RP, Muhlfeld CC, Waples RS, Luikart G. Assessments of species’ vulnerability to climate change: from pseudo to science. Biodivers Conserv. 2016; 1–7.

[3] Monahan WB, Fisichelli NA. Climate exposure of US national parks in a new era of change. PLoS ONE. 2014;9: e101302 doi: 10.1371/journal.pone.0101302 24988483 10.1371/journal.pone.0101302 PMC4079655

[4] Fisichelli NA, Schuurman GW, Hoffman CH. Is ‘resilience’ maladaptive? Towards an accurate lexicon for climate change adaptation. Environ Manage. 2016;57: 753–758. doi: 10.1007/s00267-015-0650-6 26721473 10.1007/s00267-015-0650-6 PMC4785211

[5] Langdon JG, Lawler JJ. Assessing the impacts of projected climate change on biodiversity in the protected areas of western North America. Ecosphere. 2015;6: art87.

[6] U.S. Fish and Wildlife Service. 2011 National Survey of Fishing, Hunting, and Wildlife-Associated Recreation National Overview. 2012. http://digitalmedia.fws.gov/cdm/ref/collection/document/id/859

[7] Pearce-Higgins J, Green RE. Birds and Climate Change: Impacts and Conservation Responses. Cambridge, UK: Cambridge University Press; 2014.

[8] McKinnon L, Picotin M, Bolduc E, Juillet C, Bêty J. Timing of breeding, peak food availability, and effects of mismatch on chick growth in birds nesting in the High Arctic. Can J Zool. 2012;90: 961–971.

[9] Gregory RD, Willis SG, Jiguet F, Voříšek P, Klvaňová A, van Strien A, et al An indicator of the impact of climatic change on European bird populations. PLoS ONE. 2009;4: e4678 doi: 10.1371/journal.pone.0004678 19259270 10.1371/journal.pone.0004678 PMC2649536

[10] Devictor V, van Swaay C, Brereton T, Chamberlain D, Heliölä J, Herrando S, et al Differences in the climatic debts of birds and butterflies at a continental scale. Nat Clim Chang. 2012;2: 121–124.

[11] Peterson AT, Navarro-Sigüenza AG, Martínez-Meyer E, Cuervo-Robayo AP, Berlanga H, Soberón J. Twentieth century turnover of Mexican endemic avifaunas: landscape change versus climate drivers. Sci Adv. 2015;1: e1400071 doi: 10.1126/sciadv.1400071 26601171 10.1126/sciadv.1400071 PMC4640638

[12] Bateman BL, Pidgeon AM, Radeloff VC, Allstadt AJ, Akçakaya HR, Thogmartin WE, et al The importance of range edges for an irruptive species during extreme weather events. Landscape Ecol. 2015;30: 1095–1110.

[13] McKechnie AE, Wolf BO. Climate change increases the likelihood of catastrophic avian mortality events during extreme heat waves. Biol Lett. 2010;6: 253–256. doi: 10.1098/rsbl.2009.0702 19793742 10.1098/rsbl.2009.0702 PMC2865035

[14] Pacifici M, Visconti P, Butchart SHM, Watson JEM, Cassola FM, Rondinini C. Species’ traits influenced their response to recent climate change. Nat Clim Chang. 2017;7: 205–208.

[15] Wiens JJ. Climate-related local extinctions are already widespread among plant and animal species. PLoS Biol. 2016;14: e2001104 doi: 10.1371/journal.pbio.2001104 27930674 10.1371/journal.pbio.2001104 PMC5147797

[16] Tingley MW, Koo MS, Moritz C, Rush AC, Beissinger SR. The push and pull of climate change causes heterogeneous shifts in avian elevational ranges. Glob Change Biol. 2012;18: 3279–3290.

[17] Zuckerberg B, Woods AM, Porter WF. Poleward shifts in breeding bird distributions in New York State. Glob Chang Biol. 2009;15: 1866–1883.

[18] Princé K, Zuckerberg B. Climate change in our backyards: the reshuffling of North America’s winter bird communities. Glob Chang Biol. 2015;21: 572–585. 25322929 10.1111/gcb.12740

[19] Bateman BL, Pidgeon AM, Radeloff VC, VanDerWal J, Thogmartin WE, Vavrus SJ, et al The pace of past climate change vs. potential bird distributions and land use in the United States. Glob Change Biol. 2016;22: 1130–1144. 10.1111/gcb.13154 26691721

[20] Foden WB, Butchart SHM, Stuart SN, Vié J-C, Akçakaya HR, Angulo A, et al Identifying the world’s most climate change vulnerable species: a systematic trait-based assessment of all birds, amphibians and corals. PLoS ONE. 2013;8: e65427 doi: 10.1371/journal.pone.0065427 23950785 10.1371/journal.pone.0065427 PMC3680427

[21] Langham GM, Schuetz JG, Distler T, Soykan CU, Wilsey CB. Conservation status of North American birds in the face of future climate change. PLoS ONE. 2015;10: e0135350 doi: 10.1371/journal.pone.0135350 26333202 10.1371/journal.pone.0135350 PMC4558014

[22] Culp LA, Cohen EB, Scarpignato AL, Thogmartin WE, Marra PP. Full annual cycle climate change vulnerability assessment for migratory birds. Ecosphere. 2017;8: e01565.

[23] Gardali T, Seavy NE, DiGaudio RT, Comrack LA. A climate change vulnerability assessment of California’s at-risk birds. PLoS ONE. 2012;7: e29507 doi: 10.1371/journal.pone.0029507 22396726 10.1371/journal.pone.0029507 PMC3292547

[24] Peterson AT, Soberón J, Pearson RG, Anderson RP, Martínez-Meyer E, Nakamura M, et al Ecological Niches and Geographic Distributions. Princeton, N.J: Princeton University Press; 2011 328 p.

[25] Anderson RP. A framework for using niche models to estimate impacts of climate change on species distributions. Ann N Y Acad Sci. 2013;1297: 8–28. 25098379 10.1111/nyas.12264

[26] VanDerWal J, Shoo LP, Johnson CN, Williams SE. Abundance and the environmental niche: environmental suitability estimated from niche models predicts the upper limit of local abundance. Am Nat. 2009;174: 282–291. doi: 10.1086/600087 19519279 10.1086/600087

[27] Wade AA, Theobald DM, Laituri MJ. A multi-scale assessment of local and contextual threats to existing and potential U.S. protected areas. Landsc Urban Plan. 2011;101: 215–227.

[28] Stephens PA, Mason LR, Green RE, Gregory RD, Sauer JR, Alison J, et al Consistent response of bird populations to climate change on two continents. Science. 2016;352: 84–87. doi: 10.1126/science.aac4858 27034371 10.1126/science.aac4858

[29] Distler T, Schuetz JG, Velásquez-Tibatá J, Langham GM. Stacked species distribution models and macroecological models provide congruent projections of avian species richness under climate change. J Biogeogr. 2015;42: 976–988.

[30] Sauer JR, Link WA, Fallon JE, Pardieck KL, Ziolkowski DJ. The North American Breeding Bird Survey 1966–2011: summary analysis and species accounts. North American Fauna. 2013;79: 1–32.

[31] Soykan CU, Sauer J, Schuetz JG, LeBaron GS, Dale K, Langham GM. Population trends for North American winter birds based on hierarchical models. Ecosphere. 2016;7: e01351.

[32] Pachauri RK, Mayer L, editors. Climate change 2014: synthesis report. Geneva, Switzerland: Intergovernmental Panel on Climate Change; 2015 151 p.

[33] Knutti R, Masson D, Gettelman A. Climate model genealogy: Generation CMIP5 and how we got there. Geophys Res Lett. 2013;40: 1194–1199.

[34] Allouche O, Tsoar A, Kadmon R. Assessing the accuracy of species distribution models: prevalence, kappa and the true skill statistic (TSS). J Appl Ecol. 2006;43: 1223–1232.

[35] Freeman EA, Moisen GG. A comparison of the performance of threshold criteria for binary classification in terms of predicted prevalence and kappa. Ecol Modell. 2008;217: 48–58.

[36] Oksanen J, Blanchet FG, Friendly M, Kindt R, Legendre P, McGlinn D, et al. vegan: Community Ecology Package. 2017. https://cran.r-project.org/web/packages/vegan/index.html

[37] Hole DG, Huntley B, Arinaitwe J, Butchart SHM, Collingham YC, Fishpool LDC, et al Toward a management framework for networks of protected areas in the face of climate change: management of protected-area networks. Conserv Biol. 2011;25: 305–315. 21284728 10.1111/j.1523-1739.2010.01633.x

[38] R Core Team. R: a language and environment for statistical computing. Vienna, Austria: R Foundation for Statistical Computing; 2017 http://www.R-project.org/

[39] Hellmann F, Alkemade R, Knol OM. Dispersal based climate change sensitivity scores for European species. Ecol Indic. 2016;71: 41–46.

[40] Strong C, Zuckerberg B, Betancourt JL, Koenig WD. Climatic dipoles drive two principal modes of North American boreal bird irruption. PNAS. 2015;112: e2795–2802. doi: 10.1073/pnas.1418414112 25964328 10.1073/pnas.1418414112 PMC4450426

[41] Stralberg D, Bayne EM, Cumming SG, Sólymos P, Song SJ, Schmiegelow FKA. Conservation of future boreal forest bird communities considering lags in vegetation response to climate change: a modified refugia approach. Diversity Distrib. 2015;21: 1112–1128.

[42] Charmantier A, Gienapp P. Climate change and timing of avian breeding and migration: evolutionary versus plastic changes. Evol Appl. 2014;7: 15–28. 24454545 10.1111/eva.12126 PMC3894895

[43] Wong BBM, Candolin U. Behavioral responses to changing environments. Behav Ecol. 2015;26: 665–673.

[44] Martin TE. Abiotic vs. biotic influences on habitat selection of coexisting species: climate change impacts? Ecology. 2001;82: 175–188.

[45] Gillings S, Balmer DE, Fuller RJ. Directionality of recent bird distribution shifts and climate change in Great Britain. Glob Chang Biol. 2015;21: 2155–2168. 25482202 10.1111/gcb.12823

[46] Hitch AT, Leberg PL. Breeding distributions of North American bird species moving north as a result of climate change. Conserv Biol. 2007;21: 534–539. 17391203 10.1111/j.1523-1739.2006.00609.x

[47] Poole A. Birds of North America Online. Cornell Laboratory of Ornithology, Ithaca 2005 https://birdsna.org/

[48] Currie DJ, Venne S. Climate change is not a major driver of shifts in the geographical distributions of North American birds. Glob Ecol Biogeogr. 2016;26: 333–346.

[49] Hartmann DL, Klein Tank AMG, Rusticucci M, Alexander LV, Brönnimann S, Charabi Y, et al. Observations: Atmosphere and Surface. In: Stocker TF, Qin D, Plattner G-K, Tignor M, Allen SK, Boschung A, et al., editors. Climate Change 2013: The Physical Science Basis Contribution of Working Group I to the Fifth Assessment Report of the Intergovernmental Panel on Climate Change. Cambridge, United Kingdom and New York, NY, USA: Cambridge University Press; 2013.

[50] Niven DK, Butcher GS. Northward shifts in the abundance of North American birds in early winter: a response to warmer winter temperatures? Audubon; 2009. http://www.audubon.org/sites/default/files/documents/report.pdf

[51] Visser ME, Perdeck AC, Balen V, Johan H, Both C. Climate change leads to decreasing bird migration distances. Glob Chang Biol. 2009;15: 1859–1865.

[52] Alerstam T, Hedenström A. The development of bird migration theory. J Avian Biol. 1998;29: 343–369.

[53] Pulido F, Berthold P. Current selection for lower migratory activity will drive the evolution of residency in a migratory bird population. PNAS. 2010;107: 7341–7346. doi: 10.1073/pnas.0910361107 20368446 10.1073/pnas.0910361107 PMC2867690

[54] Wuebbles D, Meehl G, Hayhoe K, Karl TR, Kunkel K, Santer B, et al CMIP5 climate model analyses: climate extremes in the United States. Bull Amer Meteor Soc. 2013;95: 571–583.

[55] Mascioli NR, Previdi M, Fiore AM, Ting M. Timing and seasonality of the United States ‘warming hole’. Environ Res Lett. 2017;12: 034008.

[56] Loarie SR, Duffy PB, Hamilton H, Asner GP, Field CB, Ackerly DD. The velocity of climate change. Nature. 2009;462: 1052–1055. doi: 10.1038/nature08649 20033047 10.1038/nature08649

[57] Carroll C, Lawler JJ, Roberts DR, Hamann A. Biotic and climatic velocity identify contrasting areas of vulnerability to climate change. PLoS ONE. 2015;10: e0140486 doi: 10.1371/journal.pone.0140486 26466364 10.1371/journal.pone.0140486 PMC4605713

[58] Ordonez A, Martinuzzi S, Radeloff VC, Williams JW. Combined speeds of climate and land-use change of the conterminous US until 2050. Nat Clim Chang. 2014;4: 811–816.

[59] McGuire JL, Lawler JJ, McRae BH, Nuñez TA, Theobald DM. Achieving climate connectivity in a fragmented landscape. PNAS. 2016;113: 7195–7200. doi: 10.1073/pnas.1602817113 27298349 10.1073/pnas.1602817113 PMC4932962

[60] Rodrigues ASL, Akcakaya HR, Andelman SJ, Bakarr MI, Boitani L, Brooks TM, et al Global gap analysis: priority regions for expanding the global protected-area network. BioScience. 2004;54: 1092–1100.

[61] Lawler JJ. Climate change adaptation strategies for resource management and conservation planning. Ann N Y Acad Sci. 2009;1162: 79–98. 19432646 10.1111/j.1749-6632.2009.04147.x

[62] Kostyack J, Lawler JJ, Goble DD, Olden JD, Scott JM. Beyond reserves and corridors: policy solutions to facilitate the movement of plants and animals in a changing climate. BioScience. 2011;61: 713–719.

[63] Millar CI, Stephenson NL, Stephens SL. Climate change and forests of the future: managing in the face of uncertainty. Ecol Appl. 2007;17: 2145–2151. 18213958 10.1890/06-1715.1

[64] Fancy SG, Gross JE, Carter SL. Monitoring the condition of natural resources in US national parks. Environ Monit Assess. 2009;151: 161–174. doi: 10.1007/s10661-008-0257-y 18509737 10.1007/s10661-008-0257-y

[65] Bartuszevige AM, Taylor K, Daniels A, Carter MF. Landscape design: integrating ecological, social, and economic considerations into conservation planning. Wildl Soc Bull. 2016;40: 411–422.

[66] Zarnetske PL, Skelly DK, Urban MC. Biotic multipliers of climate change. Science. 2012;336: 1516–1518. doi: 10.1126/science.1222732 22723403 10.1126/science.1222732

[67] Estrada A, Morales-Castilla I, Meireles C, Caplat P, Early R. Equipped to cope with climate change: traits associated with range filling across European taxa. Ecography. 2017.

[68] Beever EA, O’Leary J, Mengelt C, West JM, Julius S, Green N, et al Improving conservation outcomes with a new paradigm for understanding species’ fundamental and realized adaptive capacity. Conserv Lett. 2015;9: 131–137.

[69] Bancroft BA, Lawler JJ, Schumaker NH. Weighing the relative potential impacts of climate change and land-use change on an endangered bird. Ecol Evol. 2016;6: 4468–4477. 27386089 10.1002/ece3.2204 PMC4930994

[70] Elith J, Kearney M, Phillips S. The art of modelling range-shifting species. Methods Ecol Evol. 2010;1: 330–342.

[71] Bahn V, McGill BJ. Testing the predictive performance of distribution models. Oikos. 2013;122: 321–331.

[72] BirdLife International, National Audubon Society, editors. The messengers: what birds tell us about threats from climate change and solutions for nature and people. Cambridge, UK: BirdLife International; 2015 71 p.

[73] Colwell R, Avery S, Berger J, Davis GE, Hamilton H, Lovejoy T, et al Revisiting Leopold: resource stewardship in the national parks. PARKS. 2014;20: 15–24.

