# Supplementary material for: Projected avifaunal responses to climate change across the U.S. National Park System
Source: PLoS One. 2018 Mar 21;13(3):e0190557. doi: 10.1371/journal.pone.0190557 (PMC5862404; doi:10.1371/journal.pone.0190557)
Supplement: S1 Appendix — Results for seasonal differences, latitudinal trends, and regional differences are presented for improving, stable, and worsening species. (DOCX) [file pone.0190557.s001.docx]

**S1 Appendix. Trends of improving, stable, and worsening species across 274 U.S. national parks.** Results for seasonal differences, latitudinal trends, and regional differences are presented for improving, stable, and worsening species.

## Seasonal differences

The proportion of species in a park with improving and worsening climate suitability projections differs by season. Across the 274 parks under RCP8.5, a higher proportion of species have an improving trend in winter than in summer (*W* = 13331, *p* < 0.001; S3 Table). A higher proportion of species in parks have a worsening trend—including potential extirpations—in summer than in winter (*W* = 63016, *p* < 0.001; S3 Table). A similar proportion of species in parks will remain stable in winter and in summer (*W* = 37154, *p* = 0.8; S3 Table).

## Latitudinal trends

Under RCP8.5, the proportion of species in summer with improving climate suitability is negatively correlated with latitude in summer (*r*^2^ = 0.08, *p* < 0.001; S3 Figure) in the contiguous United States. The proportion of species with stable suitability is also negatively correlated with latitude in summer (*r^2^* = 0.15, *p* < 0.001) and winter (*r^2^* = 0.13, *p* < 0.001; S3 Figure) in the contiguous United States. The proportion of species with worsening climate suitability projections—including the proportion of potential extirpations—is positively correlated with latitude in summer (*r^2^* = 0.34, *p* < 0.001) and winter (*r^2^* = 0.13, *p* < 0.001) in the contiguous United States.

## Regional differences

The proportion of species in a park with an improving trend differs among NPS regions in summer (*F_(6, 267)_* = 6.7, *p* < 0.0001) and winter (*F_(6, 267)_* = 18.1, *p* < 0.0001). The regions with the highest proportions of species with improving trends are the Northeast in summer (0.31 ± 0.02), the National Capital in both summer (0.27 ± 0.02) and winter (0.53 ± 0.01), and the Northeast in winter (0.49 ± 0.01).

The proportion of species in a park with projected stability differs among regions in summer (*F_(6, 267)_* = 11.3, *p* < 0.001) and winter (*F_(6, 267)_* = 18.41, *p* < 0.001). In summer, the Intermountain (0.36 ± 0.01) and Southeast (0.34 ± 0.01) have the highest proportions of species with stable climate suitability, and in winter, the Southeast (0.38 ± 0.01) and Alaska (0.36 ± 0.03) have the highest proportions of species that were stable.

The proportion of species in a park with a worsening trend differs among regions in summer (*F_(6, 267)_* = 7.1, *p* < 0.0001) and winter (*F_(6, 267)_* = 4.3, *p* < 0.0001). In summer, the Midwest (0.52 ± 0.02) and Pacific West (0.52 ± 0.02) have the highest proportions of species with worsening trends, while in winter, the Midwest (0.34 ± 0.01) and the Pacific West (0.34 ± 0.02) have the highest proportions of species with worsening trends.

**Table A. Average proportions (average ± SE) of species in a park with improving, stable, and worsening trends, by RCP and season.**

| **RCP** | **Climate suitability projection** | **Summer** | **Winter** |
| --- | --- | --- | --- |
| 8.5 | Improving | 0.24 ± 0.007 | 0.39 ± 0.007 |
| 8.5 | Stable | 0.31 ± 0.006 | 0.31 ± 0.006 |
| 8.5 | Worsening | 0.45 ± 0.007 | 0.29 ± 0.006 |
| 2.6 | Improving | 0.29 ± 0.007 | 0.43 ± 0.008 |
| 2.6 | Stable | 0.32 ± 0.007 | 0.31 ± 0.007 |
| 2.6 | Worsening | 0.38 ± 0.008 | 0.26 ± 0.006 |


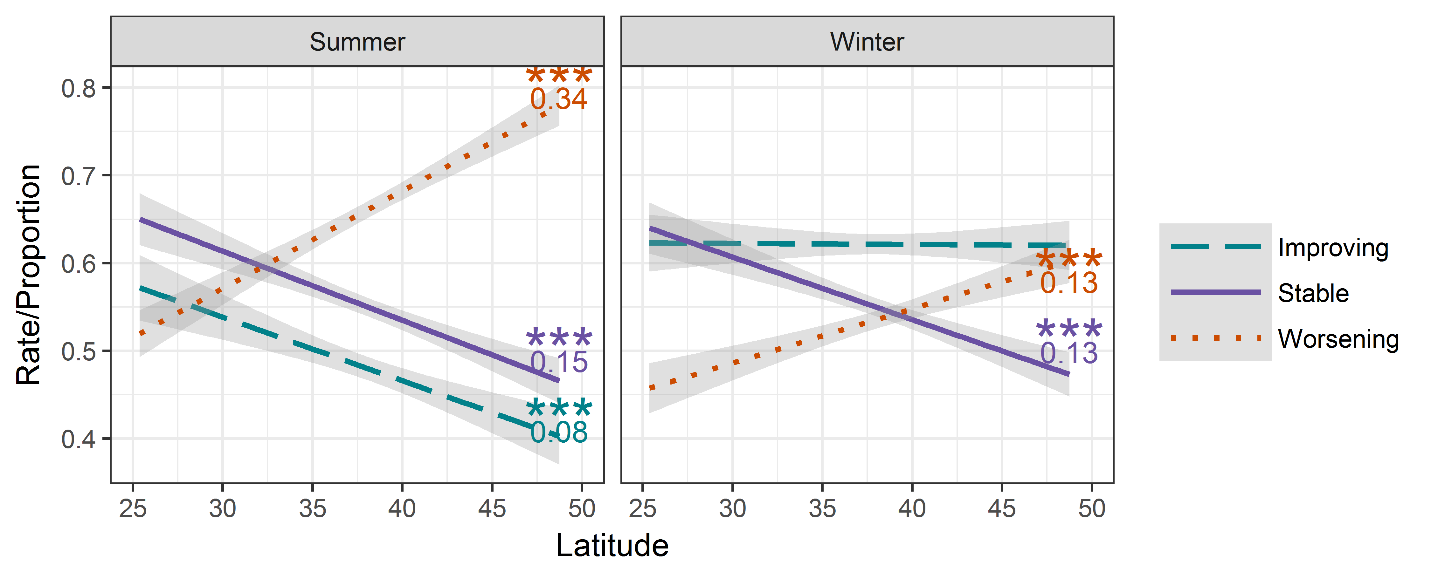


**Figure A. Relationships of the proportion of species with improving, stable, and worsening trends to latitude.** Rates/proportions between the present and mid-century under RCP8.5 in summer and winter are shown fitted to a linear regression as that improved model fit (AIC >2). Significance of the regression fit is denoted by “***” where *p* < 0.001, and *r^2^* values are shown next to each curve.
