# Supplementary figures and images for: Projected avifaunal responses to climate change across the U.S. National Park System
Source: PLoS One. 2018 Mar 21;13(3):e0190557. doi: 10.1371/journal.pone.0190557 (PMC5862404; doi:10.1371/journal.pone.0190557)

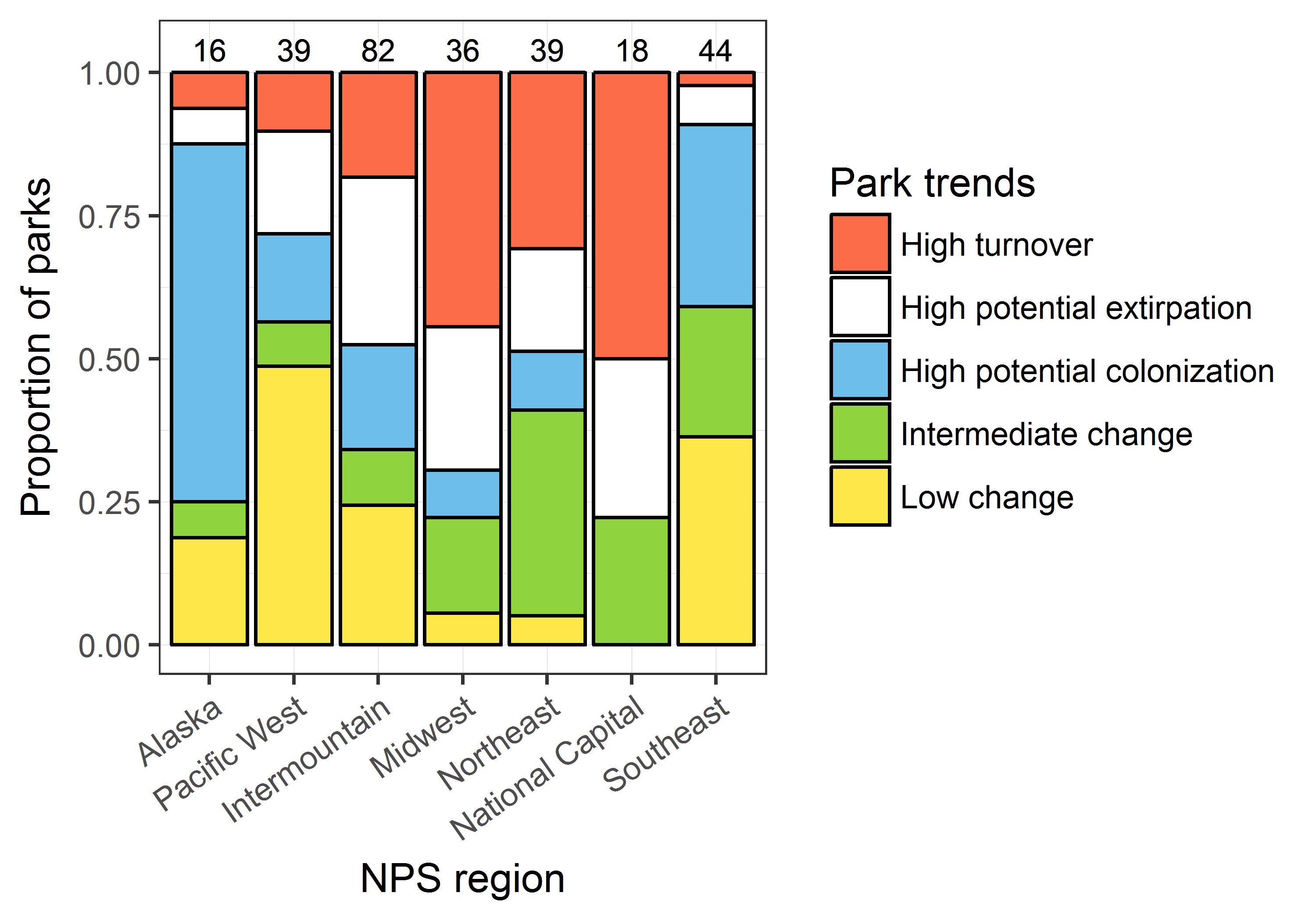

Supplement: S1 Fig — The total number of parks in each region is show above each bar. The Midwest, National Capital, and Northeast regions have the highest proportion of high-turnover parks, whereas the Pacific West and Southeast have the highest proportion of parks with low change. The Intermountain region has a more even mix of park trends. Alaska parks are primarily in the high potential colonization class. (TIF) [file pone.0190557.s002.tif]
